# Books and Magazines

**Published:** 1910-11

**Authors:** 


### Books and Magazines.

---

Hookworm Disease.—Etiology, Pathology, Diagnosis, Prognosis, Prophylaxis, and Treatment. By George Dock, A. M., M. D., Professor of the Theory and Practice of Medicine, Medical Department Tulane University of Louisiana, New Orleans, and Charles C. Bass, M. D., Instructor of Clinical Microscopy and Clinical Medicine, Medical Department Tulane University of Louisiana, New Orleans. 250 pages, royal octavo. Fifty illustrations, including one colored plate. Price, $2.50. C. V. Mosby Company, St. Louis, Publishers.
Dedicated to Charles W. Styles, Ph. D., D. S. C.

Dr. Dock's book is monumental. It is timely. It is an epitome of all that is known of hookworm to date, and no practicing physician should fail to read it. I think that Allen J. Smith is entitled to the credit of the first researches and discoveries, and not Mr. Styles. Dock knows best, I suppose.

---

Therapeutic Action of Light—A Text-Book on, including the Rho Rays, Solar and Violet Rays, Electric Arc Light, the Light Cabinet. By Gordon Eugene Rogers, M. D., formerly Demonstrator of Anatomy in the University of New York City. With original illustrations. Published by the author. Cloth; 318 pages. Price, $3. Several colored illustrations.

The last word has been said upon the treatment of pneumonia and consumption. Dr. Rogers asserts positively and unequivocally that light rays penetrate the thorax and kill the bacilli in *situ,* and that the light rays are delivered from the "Rho Lamp," of his own invention. Nuff said.

---

DIAGNOSIS OF SYPHILIS. By George E. Malsbary, M. D., Professor of Medicine, Cincinnati Polyclinic and Post-Graduate School, Author of a Text-Book on the Practice of Medicine, and Monographs on "Treatment of Tuberculosis," "The Rheumatisms," "The Septic Infections," "Meningitis," and "Cerebro-Spinal Meningitis" (in Wood's "Reference Handbook of the Medical Sciences"), Member of the Academy of Medicine of Cincinnati, the American Medical Association, the Cincinnati Obstetrical Society, etc. Bound in half Morocco. A handsome volume. A valuable addition to any library. Sent express prepaid, upon receipt of $5.00. Harvey Publishing Company, Medical Book Publishers, Merchants Building, Cincinnati, Ohio.

Did you ever fail to recognize or make a false diagnosis of syphilis and regret your failure? Physicians and surgeons, whether engaged in general or special work, are interested in the diagnosis of syphilis.

Syphilis is a subject of paramount diagnostic importance. The disease may affect practically every tissue of the body, and may resemble so many other affections that its recognition often taxes the ability of the diagnostician.

Ask any physician the most important disease from a diagnostic standpoint, and he will reply, "Syphilis." In no disease is the question of diagnosis more often at issue. How many times have you been in doubt?

No disease is more frequently improperly diagnosticated, and in no disease is improper diagnosis more dangerous—both to the patient and to the physician's reputation.

No class of society is exempt from syphilis; innocent inoculation is only too common.

Medical men are interested in the diagnosis of syphilis, not only because of the importance of the recognition of syphilis, but chiefly because syphilis so often resembles other affections.

This is a most complete work on the Diagnosis of Syphilis, that is reliable and thoroughly up to date. In this volume no part of the vast and intricate subject is neglected.

